# Heparan sulfate promotes ACE2 super-cluster assembly to enhance SARS-CoV-2-associated syncytium formation

**DOI:** 10.21203/rs.3.rs-2693563/v1

**Published:** 2023-03-28

**Authors:** Qi Zhang, Wei-Chun Tang, Eduardo Stancanelli, Eunkyung Jung, Zulfeqhar Syed, Vijayakanth Pagadala, Layla Saidi, Catherine Z. Chen, Peng Gao, Miao Xu, Ivan Pavlinov, Bing Li, Wenwei Huang, Liqiang Chen, Jian Liu, Hang Xie, Wei Zheng, Yihong Ye

**Affiliations:** The National Center for Advancing Translational Sciences; Laboratory of Pediatric and Respiratory Viral Diseases, Division of Viral Products, Office of Vaccines Research & Review, Center for Biologics Evaluation & Research, US Food & Drug Administration; University of North Carolina; University of Minnesota; National Institutes of Health; Glycan Therapeutics, LLC; National Institute of Diabetes and Digestive and Kidney Diseases; National Center for Advancing Translational Sciences; National Center for Advancing Translational Sciences; National Center for Advancing Translational Sciences; National Center for Advancing Translational Sciences; National Center for Advancing Translational Sciences; National Center for Advancing Translational Sciences; University of Minnesota; University of North Carolina; Laboratory of Pediatric and Respiratory Viral Diseases, Division of Viral Products, Office of Vaccines Research & Review, Center for Biologics Evaluation & Research, US Food & Drug Administration; National Institutes of Health; National Institutes of Health

**Keywords:** SARS-CoV-2, COVID-19, spike, ACE2, receptor clustering, syncytium, cell-cell fusion, heparan sulfate/heparan sulfate proteoglycan, Mitoxantrone, Pixantrone

## Abstract

The mechanism of syncytium formation, caused by spike-induced cell-cell fusion in severe COVID-19, is largely unclear. Here we combine chemical genetics with 4D confocal imaging to establish the cell surface heparan sulfate (HS) as a critical host factor exploited by SARS-CoV-2 to enhance spike’s fusogenic activity. HS binds spike to facilitate ACE2 clustering, generating synapse-like cell-cell contacts to promote fusion pore formation. ACE2 clustering, and thus, syncytium formation is significantly mitigated by chemical or genetic elimination of cell surface HS, while in a cell-free system consisting of purified HS, spike, and lipid-anchored ACE2, HS directly induces ACE2 clustering. Importantly, the interaction of HS with spike allosterically enables a conserved ACE2 linker in receptor clustering, which concentrates spike at the fusion site to overcome fusion-associated activity loss. This fusion-boosting mechanism can be effectively targeted by an investigational HS-binding drug, which reduces syncytium formation *in vitro* and viral infection in mice.

## Introduction

SARS-CoV-2, a single-stranded RNA virus coated with a membrane derived from host cells, injects its genetic materials into the host cytoplasm at the cell surface or following receptor-mediated endocytosis^1,2^. Both entry routes require the fusion of viral membranes with host membranes, which depends on the cell surface receptor ACE2 and the viral glycoprotein spike^3^, a single-spanning homotrimeric membrane protein^4^. During viral biogenesis, the spike undergoes a furin-mediated cleavage at the S1 site, generating two covalently linked fragments, S1 and S2^5^. The S1 fragment contains the receptor binding domain (RBD) that exists in at least two conformations: In the up conformation, the spike binds ACE2 with high affinity^6^, inducing TMPRRS2-mediated cleavage of the S2 fragment to expose a fusogenic peptide that drives the fusion of viral membranes with the plasma membrane^5,7,8^. ACE2-binding also induces receptor-mediated endocytosis, transferring viral particles to late endosomes/lysosomes where a lysosomal protease activates the spike in a similar way to promote membrane fusion^2,7,9–12^.

Intriguingly, in addition to ACE2, recent studies have established the cell surface heparan sulfate (HS) as a critical co-receptor that assists ACE2 in viral entry^9,13–16^. HS refers to a class of negative charge-enriched polysaccharides attached to the specific membrane and secretory proteins collectively termed heparan sulfate proteoglycans (HSPG)^17^. Many viruses attach to the cell surface first by binding to HS^18^. Recent studies showed that HS could bind spike directly, forming an ACE2-containing ternary complex to promote SARS-CoV-2 endocytosis^9,13,15,19,20^. The role of HS in SARS-CoV-2 infection is critical in cells with low levels of ACE2 expression^14,21,22^. Accordingly, endocytosis-mediated SARS-CoV-2 cell entry can be inhibited by HS-binding drugs or HS mimetic compounds^9,13,23^.

While ACE2-mediated viral endocytosis has been extensively studied, the mechanism underlying spike-induced membrane fusion needs to be better characterized. The spike-ACE2 interactions induce the fusion of the viral membranes with host membranes and cause virus-infected cells to fuse with ACE2-positive neighboring cells^24^. Strikingly, although cell-cell fusion rapidly dilutes the spike on the cell surface, the spike can maintain its fusogenic activity, supporting multiple rounds of fusion in a short period of time to generate multi-nuclei-containing syncytia. Large syncytia were observed in damaged lungs from postmortem COVID-19 patients^25–27^ and are thought to promote SARS-CoV-2 spreading, evasion of antibody neutralization, and disease severity^28,29^. However, how spike overcomes fusion-associated protein dilution to maintain its fusogenic activity is unclear.

Our study reveals an unexpected function for the cell surface HS in SARS-CoV-2-induced syncytium formation, explaining the spike’s long-lasting fusogenic activity. We show that upon spike ligation to ACE2, HS promotes the assembly of ACE2 into large clusters to facilitate synaptogenesis, and thus, membrane fusion. This process, which depends on a conserved linker domain in ACE2, effectively concentrates the spike at the fusion site, avoiding the loss of spike activity by fusion-associated dilution. Importantly, ACE2 clustering and syncytium formation can be targeted by an investigational drug that binds HS via specific sulfate groups, suggesting HS as a potential therapeutic target for alleviating severe COVID-19 symptoms associated with syncytium formation.

## Results

### A HS inhibitor mitigates SARS-CoV-2 infection *in vitro* and in a mouse model

**Figure 1:**
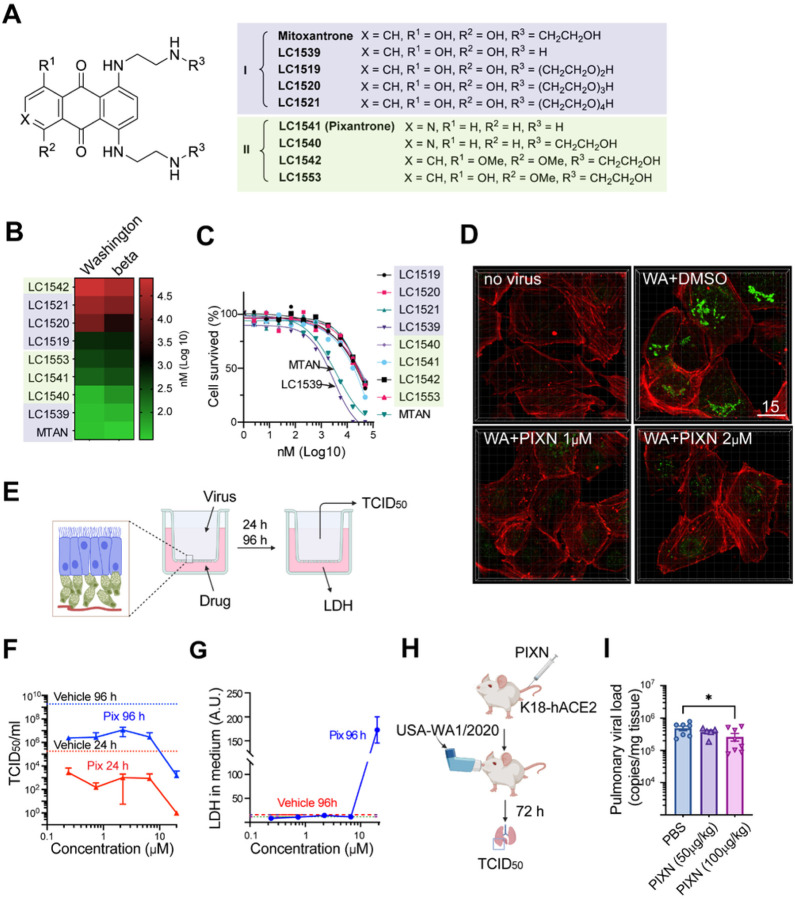
A MTAN-related drug inhibits SARS-CoV-2 infection *in vitro* and in a mouse model (A) MTAN and derivatives are classified into two groups based on structures. (B) The inhibitory activity of MTAN derivatives on the entry of pseudoviral particles coated with the spike of the indicated variants. Shown is a heat map of IC_50_ averaged from three repeats. (C) The effect of MTAN derivatives on cell viability. 293T cells were treated with the indicated chemicals for 48 h before cytotoxicity was measured. (D) PIXN inhibits the entry of USA-WA1/2020. Vero E6-TMPRSS2-T2A-ACE2 (TA6) cells were infected with live SARS-CoV-2 USA-WA1/2020 strain (WA) or mock-infected for 4 h in the presence of the indicated drugs. Cells were fixed and stained with a spike antibody in green and an actin dye in red. Scale bar, 15 μm. (E) A schematic illustration of the 3D-EpiAirway assay. (F) PIXN inhibits SARS-CoV-2 infection in the 3D EpiAirway model. TCID_50_ was determined either 24 h (red) or 96 h (blue) after the tissues were treated with PIXN and then air-infected with USA-WA1/2020 at MOI 0.1 for 1 h. The dashed lines indicate the viral titers from infected tissues without drug treatment. Error bars indicate means ± SEM. n=2. (G) PIXN does not induce significant cytotoxicity in the 3D EpiAirway model. AU, arbitrary unit. (H) The experimental scheme for *in vivo* testing of PIXN in K18-hACE2 transgenic (Tg) mice. (I) PIXN reduces SARS-CoV-2 infection in K18-hACE2 mice. Mice were pretreated with PIXN at the indicated doses followed by intranasal infection with live USA-WA1/2020. Viral load in the lung was determined 72 h post infection by qRT-PCR. Error bars indicate means ± SEM, *, p<0.05 by unpaired Mann-Whitney test.

Mitoxantrone (MTAN), an HS-binding drug, inhibits endocytosis-mediated entry of SARS-CoV-2 and other HS-dependent cargo^9,30^. However, MTAN’s cytotoxicity has limited its use as a potential antiviral drug. To overcome this problem, we synthesized a collection of derivatives (Figure 1A) and measured their cytotoxicity and antiviral activity against two pseudoviruses coated with the spike of different SARS-CoV-2 variants. MTAN contains a di-hydroxy-anthraquinone core and two symmetric arms bearing a hydroxyethyl amino group. Increasing the arm length reduced both the toxicity and antiviral activity (Figures 1B, C) while replacing the hydroxyethyl amino group with hydrogen did not affect either. Thus, modulating the arms cannot segregate the antiviral activity from cytotoxicity. By contrast, substituting several functional groups in the anthraquinone core reduced the cytotoxicity by at least one magnitude while only had a modest impact on the antiviral activity (LC1540 and LC1541) (Figures 1B, C). We chose LC1541 (also known as Pixantrone or PIXN) for further study because of its potent inhibition on spike-bearing pseudoviruses (Figure S1A), strong affinity to HS (Figure S1B), and most importantly, its confirmed safe clinical profile^31^.

Using Vero TA6 cells, a Vero E6-derived monkey epithelial cell line overexpressing human TMPRSS2 and ACE2, we found that PIXN effectively mitigated the entry of an authentic SARS-CoV-2 variant (USA-WA1/2020) (Figure 1D), which depends mainly on endocytosis (Figure S1C)^11,22^. Furthermore, in a 3D-EpiAirway system simulating human airway infection (Figure 1E)^32^, PIXN-treated samples at both 24 and 96 hours post SARS-CoV-2 infection showed a dose-dependent reduction in viral load when compared to vehicle-treated controls (Figure 1F). Meanwhile, measuring the released lactate dehydrogenase (LDH) in the medium as an indicator of cytotoxicity detected no significant cell death in PIXN-treated samples except in prolonged treatment with the highest dose (20 μM) (Figure 1G).

While numerous studies have established HS as an assisting factor for SARS-CoV-2 entry *in vitro*^19^, it is unclear whether HS affects SARS-CoV-2 infection *in vivo*. We therefore used a mouse model to further evaluate the anti-SARS-CoV-2 activity of PIXN (Figure 1H). To this end, we injected PIXN intravenously at two doses (50 μg/kg and 100 μg/kg) into K18-hACE2 transgenic mice expressing human ACE2^33^. We then infected the mice with live USA-WA1/2020. Seventy-two hours post-infection, we collected lung tissues and measured the viral load by qRT-PCR (Figure 1 H). We found that for animals treated with PIXN at 100 μg/kg, the viral load was reduced by ~60% compared to the control group, while at a lower dose, a minor reduction was observed (Figure 1I). These results show that PIXN, as an HS-binding drug, can mitigate SARS-CoV-2 infection *in vivo*, although the activity is weaker than that seen in cell-based assays. The low *in vivo* anti-SARS-CoV-2 activity may be due to PIXN binding to HS in non-targeting tissues, which reduces its concentration in the lung.

### PIXN and MTAN interact with HS via specific sulfate groups

**Figure 2:**
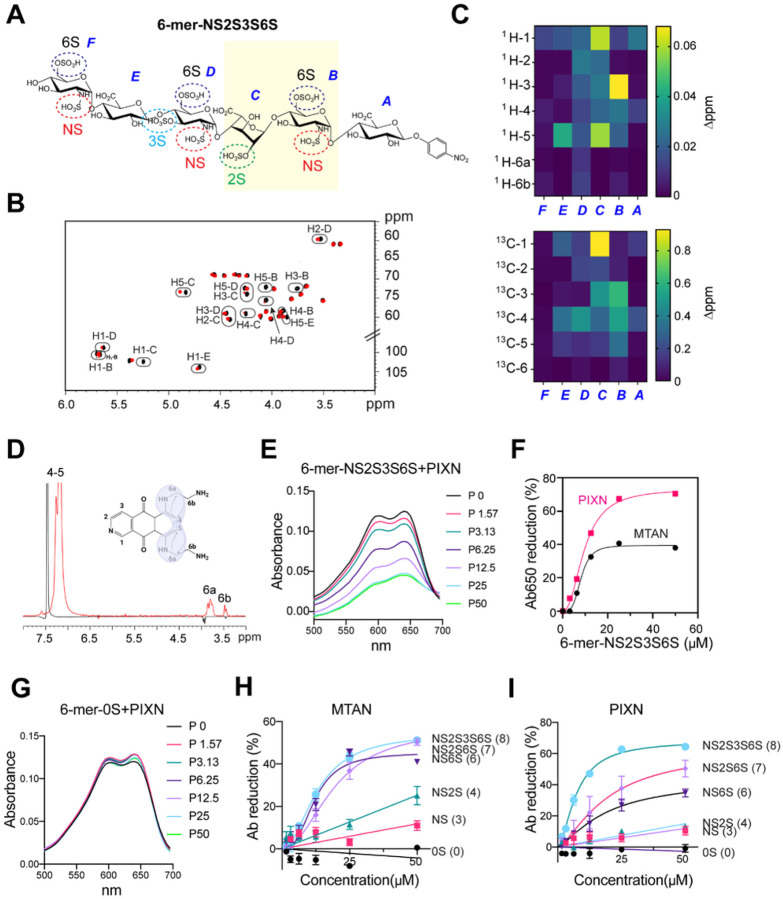
PIXN and MTAN bind distinct sulfate groups on HS (A) The chemical structure of 6-mer NS2S3S6S. This hexasaccharide bears three N-sulfate, three 6-sulfate, one 2S, and one 3S groups. The yellow shaded domain is most significantly affected by PIXN as determined by NMR. (B)^1^H, ^13^C HSQC spectrum of the 6-merNS2S3S6S. The figure shows a superposition of HSQC spectra of the free substrate in black and with addition of the ligand PIXN in red (2:1 ratio 6-mer:PXT). Peaks with notable Chemical Shift Perturbation (CSP) are circled and labelled. (C) Heat map of 6-merNS2S3S6S CSP after binding with PIXN. Absolute values of Δppm for both ^1^H (left) and ^13^C (right) were plotted. The central part of 6-merNS2S3S6S shows higher valus of CSP suggesting that the interaction takes place only with the inner residues of the oligosaccharide. (D) 1D-NOE comparison of free PIXN (black) and upon binding (red) suggests a modification in the spatial proximity of protons surrounding the aromatic amino groups when the interaction with 6-merNS2S3S6S occurs. (E) 6-mer NS2S3S6S binding changes the absorption spectra of PIXN. The numbers indicate drug concentrations (μM). (F) Both PIXN and MTAN HS 6-mer NS2S3S6S. PIXN or MTAN (50 μM) was incubated with 6-mer NS2S3S6S as indicated. The changes in Ab_650_ were plotted. (G) As in (E) except that a HS 6-mer without any sulfate groups was used. (H, I) The differential contribution of the sulfate groups to MTAN (H) and PIXN (I) binding. As in (F), except that HS 6-mer analogs bearing different number of sulfate groups (indicated by the numbers) were used. Error bars indicate means ± SEM. n=3.

We next used a synthetic HS hexasaccharide (the six sugar moieties are designated as A to F, respectively) with defined sulfate groups (named 6-mer-NS2S3S6S) to characterize how PIXN and MTAN interact with HS (Figure 2A) because the HS 6-mer binds these drugs similarly as longer heterogenous HS chains (see below). We first used NMR to measure chemical shift perturbations of HS 6-mer NS2S3S6S caused by PIXN. MTAN was omitted from this experiment because of its self-aggregation property at the concentration required for the NMR study. The result showed that most spectral changes happened on the sugar moieties B-D, centering around the C-3 position in N-sulfo-D-glucosamine B and the anomeric position in glucuronic acid C (Figure 2B, C). Reciprocal titration of HS 6-mer NS2S3S6S to PIXN showed that the two -NH groups on the arms were most significantly affected (Figure 2D). These results suggest that HS might use specific sulfate groups (e.g., 2S, NS, and 6S) in the sugar moieties B-D to interact with the -NH groups of PIXN. This model is consistent with the observation that the -NH groups are conserved in MTAN.

To validate the NMR results, we used a binding assay based on the fact that both MTAN and PIXN absorb light at 650 nm^9^ and that the interaction of these drugs with HS or HS 6-mer NS2S3S6S resulted in a dose-dependent reduction in Ab^650^ (Figure 2E, Figure S2A). Plotting the change in Ab^650^ over HS concentrations suggested that PIXN bound to 6-mer-NS2S3S6S with a similar affinity as MTAN. However, the maximum binding for PIXN was about two-fold higher than that of MTAN (Figure 2F). As expected, an HS 6-mer analog bearing no sulfate group (6-mer-0S) did not interact with either PIXN or MTAN (Figure 2G and Figure S2B), demonstrating a sulfate-dependent interaction with these drugs.

To identify the sulfate group(s) involved in drug binding, we performed a binding study using synthetic HS 6-mers containing only given types of sulfate groups (Figure S2C). The results showed that the drug binding depends on both sulfate number and position (Figure 2H, I); Low binding was generally detected for HS 6-mer carrying three NS groups. For MTAN, the addition of three 6S groups (NS6S) increased the affinity dramatically, while having additional 2S and 3S groups (NS6S2S or NS2S3S6S) did not further improve MTAN binding (Figure 2H). By contrast, 6-mer NS6S only had a modestly increased affinity for PIXN compared to 6-mer NS, while adding 2S and 3S maximized the affinity to PIXN (Figure 2I). Thus, while PIXN and MTAN both bind HS via sulfate groups, they have different preferences for sulfate position.

### Pharmacological inhibition of HS mitigates SARS-CoV-2-induced cell-cell fusion

**Figure 3:**
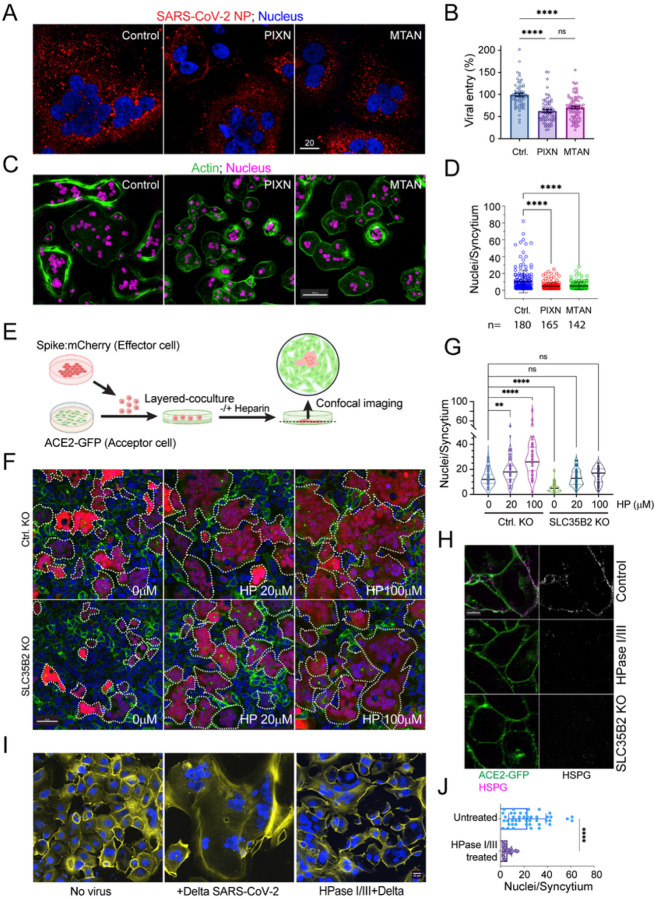
The cell surface HS promotes spike-induced cell-cell fusion (A, B) MTAN and PIXN reduce the entry of the SARS-CoV-2 Delta variant. (A) Vero TA6 cells treated with the indicated drugs were infected with live Delta variant at MOI of 0.1 for 4 h. Cells were fixed and stained with an anti-NP antibody that recognizes the viral nucleocapsid protein (red) and a Hoechst dye (blue). Scale bar, 20 μm. (B) Quantification of the NP signals in individual cells from two independent experiments each with two technical repeats. ****, p<0.0001 by unpaired student’s t-test, n=4, ns, not significant. (C, D) MTAN and PIXN reduce the size of SARS-CoV-2-induced syncytia. (C) Vero TA6 cells infected with live Delta variant at MOI of 0.1 in the absence (control) or presence of the indicated drugs for 6 h. Cells were stained by an actin dye (green) and Hoechst (magenta). (D) Quantification of the syncytium size in (C). The n values indicate the number of syncytium counted. Error bars indicate means ± SD, ****, p<0.0001 by unpaired student’s t-test. n=4. (E) A schematic illustration of the optimized cell-cell fusion assay. (F, G) SLC35B2 knockout (KO) inhibits spike-induced cell-cell fusion, which is rescued by heparin. (F) WT (Ctrl.) and SLC35B2 CRISPR KO cells expressing ACE2-GFP in a monolayer were incubated with spike/mCherry cells in the absence or presence of heparin (HP). Cells were imaged after 1 h of incubation. Dashed lines indicate syncytia. (G) Quantification of the experiments. **, p<0.01; ****, p<0.0001 by one-way ANOVA. n=3 independent repeats. (H) Super-charged CFP staining validates the lack of cell surface HS by either heparinase I/III treatment or SLC35B2 KO. (I, J) Heparinase I/III treatment inhibits Delta-induced cell-cell fusion. (I) Vero TA6 cells were mocked treated or treated with heparinase I/III for 3 h prior to infection with live SARS-CoV-2 Delta variant (MOI=0.5). Cells were fixed and stained with an actin dye (yellow) and Hoechst (blue). (J) Quantification of (I). ****, p<0.0001 by unpaired student’s t-test. n=3 independent repeats.

Having established PIXN and MTAN as HS-binding drugs that block endocytosis-mediated entry of SARS-CoV-2, we tested whether these drugs inhibited SARS-CoV-2 entry via fusion at the plasma membrane. We chose the Delta variant (B.1.617.2) because recent studies suggested that the spike of this variant has the most robust membrane fusion-stimulating activity compared to spikes of other variants^34^. Consistent with this view, when Vero TA6 cells infected with the Delta variant were stained with an antibody against the SARS-CoV-2 nucelocapsid protein (NP), we detected viral particles as small puncta throughout the cytoplasm (Figure 3A). Cells treated with either PIXN or MTAN during infection still contained NP-positive puncta. However, the intensity was reduced by ~50% (Figure 3A, B). Thus, both PIXN and MTAN can inhibit Delta entry via the plasma membrane.

As expected from the robust fusogenic activity of the Delta spike, we observed many multinuclear syncytia in cells infected with the Delta variant (Figure 3C, D). Presumably, the fusion of the viral envelope with the plasma membrane redistributes the spike to the cell surface, causing infected cells to fuse with nearby cells. Consistent with this notion, infection with the endosome-bound USA-WA1/2020 generated few syncytia under the same conditions (Figure S3A). In Delta-infected samples, some syncytia are giant, containing up to 80 nuclei. Their formation requires at least seven rounds of fusion. Given the low MOI used, it is surprising that the limited spike molecules transferred from the viral particles can maintain such high fusogenic activity, particularly as each round of fusion increases the cell surface area, which further reduces the spike concentration. Interestingly, when cells infected with the Delta variant were treated with PIXN or MTAN, syncytium size was much reduced, with most syncytia containing only 4–6 nuclei. These findings suggest a fusion-boosting mechanism that helps overcome fusion-associated fusogen dilution, which is sensitive to HS-binding drugs.

### HS promotes spike-induced syncytium formation

To elucidate the role of HS in spike-induced cell-cell fusion, we optimized a co-culture-based fusion assay (Figure 3E). We co-transfected 293T cells with the Delta spike- and mCherry-expressing plasmids (Effector) and then added these cells in suspension to a monolayer of 293T cells stably expressing ACE2-GFP (Acceptor). Spike-expressing cells were round and mostly mCherry-positive, but after fusing with ACE2 cells, they attached to the surface and flattened into irregular shapes. After incubation, unfused cells were removed by washing. The fusion efficiency could be monitored by counting the number of nuclei per syncytium under fluorescence microscopy. In this assay, syncytium formation strictly depends on the spike in effector cells and ACE2 in acceptor cells^35^. Additionally, both PIXN and MTAN inhibited syncytium formation in this assay (Figure S3B, C)^35^, suggesting that the assay recapitulates virus-associated syncytium formation.

We next created HS-deficient ACE2-GFP cells by CRISPR-mediated knockout (KO) of SLC35B2, a Golgi-localized sulfate transporter essential for the sulfation of HS chains. Staining cells with a super-charged cyan fluorescent protein (CFP+) (Figure 3H), a positive charge-bearing fluorescence protein that binds HS with high affinity, confirmed the HS deficiency in the KO cells^9^. When SLC35B2 KO or control ACE2-GFP cells were co-cultured with spike/mCherry cells, syncytia formed by SLC35B2 KO cells were significantly smaller than in wild-type control despite similar levels of ACE2-GFP expression (Figure 3F, G). Notably, the cell fusion defect of SLC35B2 deficient cells was rescued entirely when heparin, a heavily sulfated HS, was added to the medium, attributing the phenotype to HS deficiency. Heparin treatment also increased the size of syncytia in wild-type (WT) cells. These results demonstrate a critical role for the cell surface HS in spike-induced cell-cell fusion, which can be substituted by unanchored heparin.

To further validate the role of HS in membrane fusion, we treated ACE2-GFP cells with heparinase I/III to remove the cell surface HS (Figure 3H). Like SCL35B2 KO cells, heparinase-treated ACE2-GFP cells, when co-cultured with spike/mCherry cells, produced syncytia of smaller sizes (Figure S3D, E). Likewise, when heparinase-treated Vero TA6 cells were infected with the Delta strain, we found that heparinase treatment significantly reduced the syncytium size (Figure 3I, J). Because removing HS from acceptor cells alone is sufficient to mitigate cell-cell fusion and because high concentrations of heparin are required to restore the fusion activity in SLC35B2 KO cells, it appears that HS acts most effectively when positioned *in cis* to ACE2-containing membranes (see discussion).

### HS enables synapse-like cell-cell contacts in spike-mediated syncytium formation

**Figure 4:**
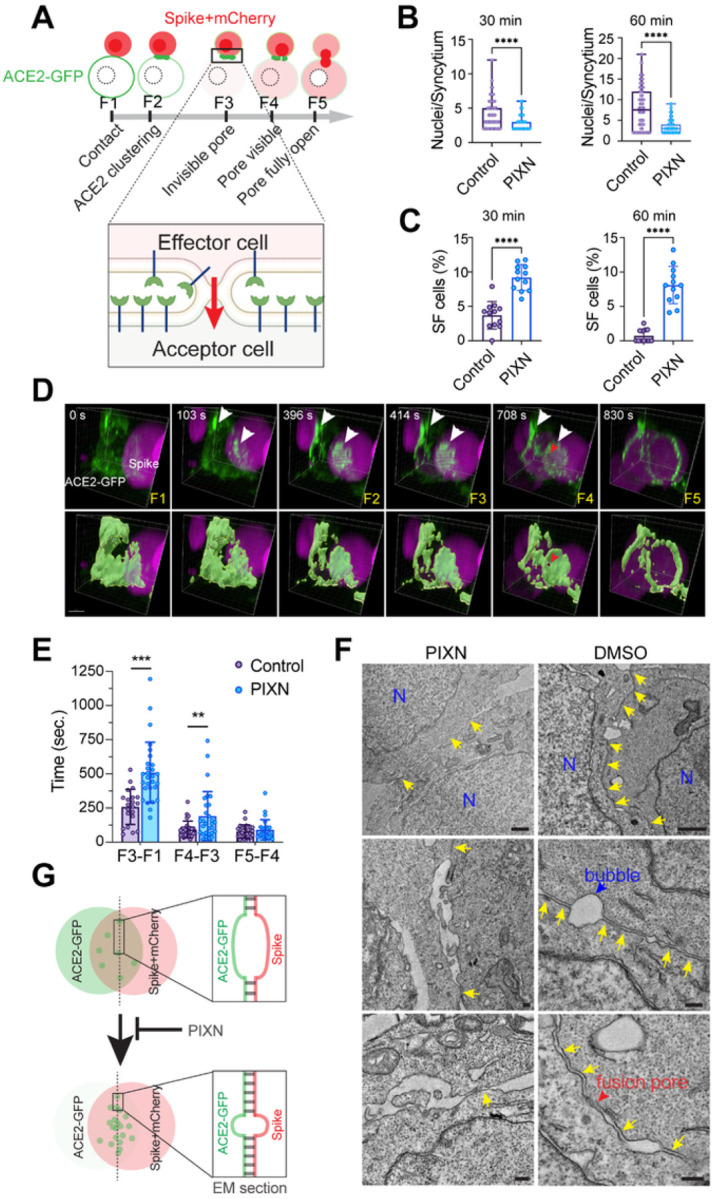
HS promotes fusion pore formation to enhance spike-induced cell-cell fusion (A) A schematic illustration of the cell-cell fusion process. F1-F5 indicated different stages determined by 4D imaging. (B, C) PIXN inhibits spike-induced cell-cell fusion at a semi-fusion stage. Spike-induced cell-cell fusion was performed in the absence or presence of 1 μM PIXN for 30- or 60-min. Cells were fixed and stained with Hoechst to label nuclei. (B) The number of nuclei per syncytium and (C) of semi-fusion (SF) cells was counted. Representative images are presented in Figure S4A. Error bars in (C) indicate means ± SD, n=2. ****, p<0.0001 by unpaired student t-test. (D) 4D confocal imaging of spike-induced cell-cell fusion. Frames were selected to represent the different stages in (A). The bottom panels show surface-rendered ACE2-GFP signals. White arrowheads indicate ACE2-GFP clusters. The red arrowhead labels a visible fusion pore. Scale bar, 5 μm. (E) PIXN delays the semi-fusion stage and the appearance of visible fusion pores. Shown are the times between the indicated fusion stages measured by 4D imaging. Error bars indicate means ± SD, **, p<0.01, ***, p<0.001 by unpaired student’s t-test. (F) PIXN disrupts the synapse-like cell-cell contacts between spike and ACE2-GFP cells. Shown are representative EM images of co-cultured cells untreated or treated with PIXN. N, nucleus; arrows show juxtaposed membranes at the contact sites. The red arrow shows an example of disrupted membranes due to fusion. Scale bars, 1 μm for top panels, 200nm for other panels. (G) A schematic diagram of the result in (F).

To fuse with acceptor cells, an effector cell needs to go through the following conceptual steps: 1) forming cell-cell contact, 2) generating a small fusion pore, 3) the pore widening into a significant gap, and 4) complete fusion of the two cells (Figure 4A). We used the co-culture assay to narrow down the steps affected by PIXN. Specifically, in addition to measuring the syncytium size (Figure 4B), we counted the number of mononuclear cells that have both mCherry and ACE2-GFP (Figure 4C). These cells are in a semi-fusion state (F3 in Figure 4A) in which a small fusion pore has formed, allowing mCherry to enter the ACE2-GFP cell, but the membrane boundary between the two cells remains largely intact. In vehicle-treated reactions, ~4% of ACE2-GFP cells were detected in this semi-fusion state after 30 min incubation with spike/mCherry cells. In contrast, in PIXN-treated conditions, the number of semi-fusion cells increased to ~9% (Figure 4C, Figure S4A). After further incubation, semi-fusion cells were almost undetectable in the control reaction because most cells had completed at least one round of fusion. However, in the presence of PIXN, ~8% of ACE2 cells remained in the semi-fusion state. These results suggest that PIXN delays the opening of the fusion pore to inhibit cell-cell fusion.

To elucidate the precise step that involves HS, we used 4D live cell confocal microscopy to monitor the effect of PIXN on cell-cell fusion in real-time. Analyses of randomly selected fusion events confirmed that PIXN treatment delayed both the entry of mCherry into ACE2-GFP cells and the appearance of a visible pore. However, once a visible pore was formed, the pore widening was unaffected (Figure 4C, D). These results suggest that HS facilitates fusion pore formation.

How does HS inhibition delay the formation of fusion pores? Since biochemical studies showed that neither PIXN nor MTAN inhibited HS binding to the spike (Figure S4B) and that PIXN did not affect the interaction of the spike with ACE2 *in vitro* regardless of whether HS was present (Figure S4C), HS is likely dispensable for the initiation of cell-cell contact. Consistent with this notion, when spike cells contact ACE2 cells, the spike is processed by the cell surface TMPRSS2, generating an S2’ fragment and a fusion peptide. This process was also unaffected by PIXN or knockout of SLC35B2 in acceptor cells (Figure S4D, E).

Transmission electron microscopy showed that 12 min after mixing spike- and ACE2- cells, these cells form synapse-like cell-cell contacts characterized by long juxtaposed plasma membranes interrupted by bubble-like structures. The paralleled membranes, unseen when ACE2 cells were incubated with cells without spikes (Figure S4F), are only separated by a gap of less than 20 nm (Figures 4E, right panels, 4F). Importantly, we frequently observed obscure membrane boundaries at these membrane contact sites, probably caused by localized fusion events. By contrast, in PIXN-treated samples, cell-cell contacts were seen, but membranes were only held together at a few spots, leaving significant gaps in between, and fusion pores were rarely seen (Figures 4E, left panels, 4F). Thus, HS facilitates the formation of tight membrane junctions between the effector and acceptor cells in spike-induced cell-cell fusion.

### HS enhances ACE2 clustering at the fusion sites

**Figure 5:**
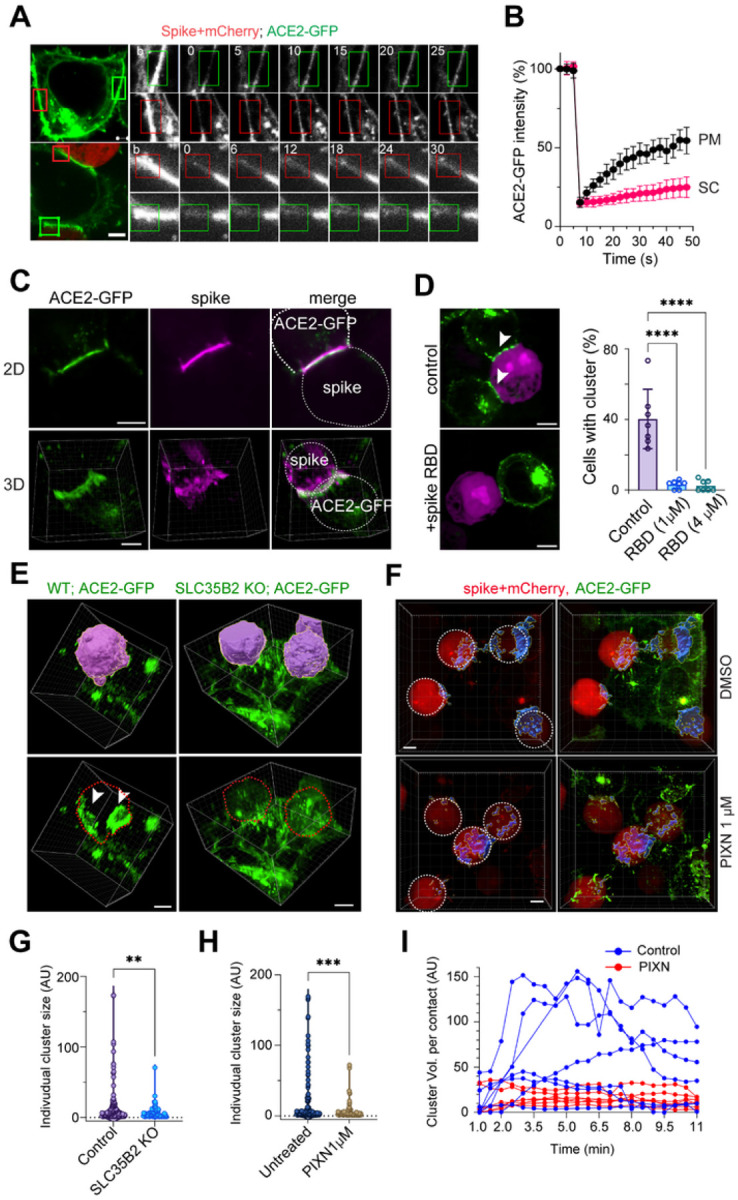
HS is required for spike-induced ACE2 super-cluster formation (A, B) ACE2 is immobile in spike-induced clusters. (A) ACE2-GFP cells (top panels) or ACE2-GFP cells incubated with spike/mCherry cells for 5 min (bottom panels) were photobleached (the box-indicated areas) and imaged by live cell confocal microscopy. Scale bars, 5 μm. (B) Quantification of the experiments in (A). In the presence of spike/mCherry cells, ACE2-GFP in super-clusters (SC) were bleached. For ACE2-GFP cells without spike/mCherry cells, ACE2-GFP in the plasma membrane (PM) was bleached. Error bars indicated means ± SD. n=8. (C) Colocalization of the spike with ACE2 in ACE2 clusters. ACE2-GFP cells were incubated spike/mCherry cells for 5 min. Cells were fixed and stained with anti-spike antibodies (magenta). Shown are two examples in 2D and 3D views, respectively. Scale bars, 5 μm. (D) Spike RBD inhibits ACE2 clustering. ACE2-GFP cells were incubated with spike/mCherry cells in the absence or presence of purified spike RBD for 10 min before imaging. Scale bars, 5 μm. The graph shows the percentage of cells containing ACE2 cluster. ****, p<0.0001 by one-way ANOVA with Dunnett’s multiple comparisons test, n=2 independent experiments. (E-H) HS is required for spike-induced ACE2 clustering. (E) Wild-type (WT) or SCL35B2 KO ACE2-GFP cells were incubated with spike/mCherry cells for 10 min before fixing and imaging. mCherry positive cells are shown in a surface-rendered view (top panels) or by the dashed lines (bottom panels). The arrowheads indicate ACE2 clusters at the cell-cell contact sites. (F) as in (E) except that WT ACE2-GFP cells were incubated with spike/mCherry cells in the absence or presence of PIXN (1 μM). ACE2-GFP clusters are shown in surface-rendered view in blue. The position of the spike/mCherry cells are indicated by the dashed lines. Scale bars, 5 μm. (G, H) Quantification of the size distribution of ACE2 clusters in (E) and (F), respectively. **, p<0.01; ***, p<0.001 by unpaired student’s t-test. n=3 independent experiments. (I) Kinetic analysis of ACE2 cluster formation in control and PIXN-treated ACE2 cells. ACE2-GFP cells incubated with spike/mCherry cells in the absence or presence of 1 μM PIXN were subject to 4D confocal imaging. The volume (Vol.) of ACE2 clusters at randomly selected cell-cell contact sites were analyzed by Imaris.

4D live cell confocal imaging revealed that within minutes following the contact of spike cells with ACE2-GFP cells, ACE2-GFP was rapidly concentrated at the cell-cell contact sites (Figure 4C, video 1), consistent with a recent report^26^. The ACE2-GFP clusters expanded over time, forming synapse-like structures with a diameter of 10–20 μm. Fluorescent recovery after photobleaching (FRAP) suggested that ACE2-GFP was almost entirely immobile in these clusters (Figures 5A, B), suggesting that ACE2 clusters are likely formed by ordered intermolecular interactions as opposed to liquid-liquid phase separation. As expected, immunostaining with spike antibodies also detected spike accumulation at the cell-cell contact sites (Figure 5C). Since recombinant spike fragments containing the receptor binding domain (RBD) could completely block ACE2 cluster formation (Figure 5D), ACE2 clustering requires spike-ACE2 interactions. Interestingly, 3D confocal imaging revealed enrichment of actin laments around the ACE2 clusters, resembling those in the immune synapse (Figure S5). Altogether, these results suggest that the interaction of the spike with ACE2 drives ACE2 into clustered super-assemblies together with the spike at the membrane contact sites. Because ACE2 clustering always precedes the entry of mCherry into the ACE2-GFP cell, ACE2 clustering must play a role in spike-induced cell-cell fusion.

To determine whether HS regulates ACE2 cluster formation, we compared the size of ACE2 clusters in WT cells to that in the SLC35B2 KO cells because the latter lack HS. When SCL35B2 KO ACE2-GFP cells were incubated with spike/mCherry cells, the size of ACE2 clusters was much smaller than those in WT ACE2-GFP cells (Figure 5E, G). Likewise, PIXN treatment of ACE2-GFP cells significantly reduced the size of ACE2 clusters (Figures 5F, H). Kinetic analysis of cluster formation by 4D confocal imaging further confirmed the ACE2 clustering defect when the cell surface HS is inhibited by PIXN (Figure 5I). These results suggest that HS is required for spike-induced ACE2 super-cluster formation.

### HS acts via a conserved ACE2 linker to promote receptor clustering and cell-cell fusion

**Figure 6:**
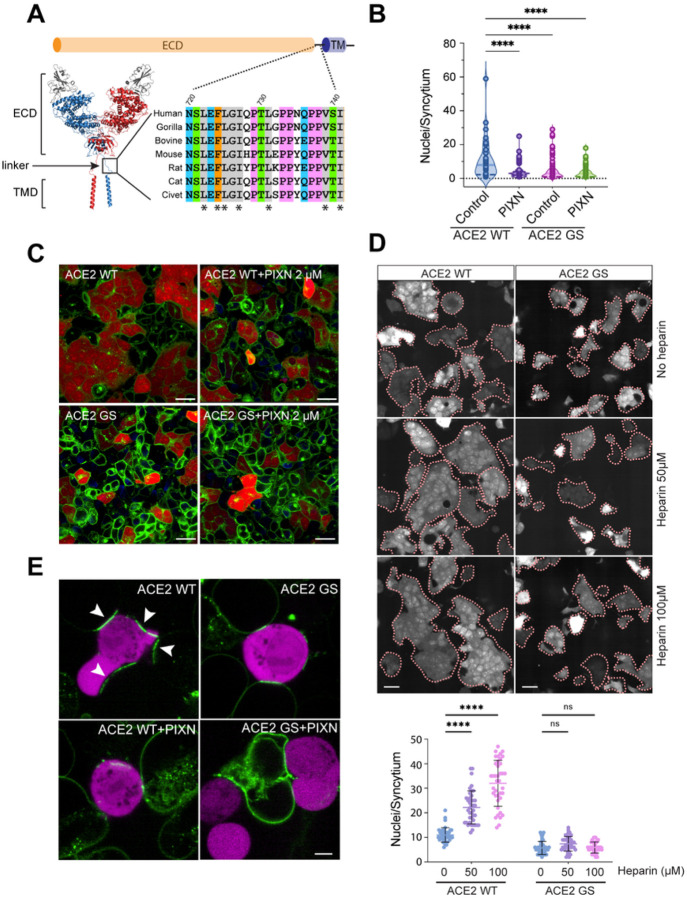
A conserved ACE2 linker mediates spike-induced receptor clustering and syncytium formation (A) A schematic view of the ACE2 structure showing a conserved extracellular linker. ECD, extracellular domain; TMD, transmembrane domain. The asterisk indicates conserved hydrophobic residues. (B, C) The ACE2 linker promotes spike-induced cell-cell fusion. Cells stably expressing WT ACE2-GFP or the ACE2-GS-GFP mutant were incubated with spike/mCherry cells in the absence (left panels) or presence of 2 μM PIXN for 60 min before imaging. Representative images are shown in (C). Scale bars, 30 μm. Quantification of the experiments is shown in (B). ****, p<0.0001 by one-way ANOVA. n=3 independent experiments. (D) Heparin-stimulated cell-cell fusion requires the ACE2 linker. WT ACE2-GFP cells or ACE2-GS-GFP cells were incubated with spike/mCherry cells in the presence heparin at the indicated concentrations for 60 min. Shown are representative images of the mCherry channel. Dashed lines indicate the boundary of the syncytia. Scale bars, 30 μm. The graph shows the quantification of the experiments. ****, p<0.0001 by one-way ANOVA. n=3 independent experiments. (E) The ACE2 linker is required for spike-induced receptor clustering. ACE2-GFP or ACE2-GS-GFP cells were incubated with spike/mCherry cells (magenta) in the absence (top panels) or presence of PIXN (1 μM) (bottom panels) for 10 min before imaging. Note that ACE2 clustering enriches ACE2 signals at cell-cell contact sites (arrowheads), depleting it from other cell surface areas in ACE2 WT-GFP cells. Scale bar, 5 μm.

As HS does not change the affinity of the spike for ACE2, we postulated that HS might induce a conformational change in ACE2, exposing a motif that drives receptor clustering. ACE2 is a single-spanning membrane protein with a large extracellular domain (ECD) for spike and ligand binding. The ECD is connected via an unstructured linker to the transmembrane domain, which mediates receptor dimerization. Intriguingly, albeit with no designated function, the unstructured linker is evolutionarily conserved (Figure 6A).

To test whether the linker segment (LS) might be critical for spike-induced cell-cell fusion, we created a 293T cell line expressing a mutant ACE2-GFP with the LS replaced by a synthetic linker bearing a glycine-serine repeat (ACE2-GS) of the same length. Immunoblotting and fluorescence imaging showed that ACE2-GS-GFP was expressed at a similar level as WT ACE2-GFP and localized to the cell surface and endocytic vesicles similarly to WT ACE2-GFP (Figures S6A, B). Furthermore, binding studies showed that ACE2-GS-GFP bound to the spike with a similar affinity as WT ACE2-GFP (Figure S6C). Nevertheless, when co-cultured with spike-expressing cells, the ACE2-GS-GFP cells had dramatically reduced fusion activity, resulting in syncytia smaller than those formed by WT ACE2-GFP cells (Figure 6B, C). Notably, while heparin stimulated the fusion of ACE2-GFP cells with spike cells, it failed to do so when ACE2-GSGFP cells were incubated with spike cells (Figure 6D), suggesting that HS acts via the ACE2 LS to promote syncytium formation. Moreover, when co-cultured with spike cells, ACE2-GS-GFP cells only formed ACE2 clusters with reduced size (Figure 6E, Figure S6D); PIXN did not further reduce the size of ACE2 clusters in ACE2-GS-GFP cells. These results suggest that the conserved LS in ACE2 is critical for HS’s function in ACE2 clustering and spike-mediated cell-cell fusion.

### HS enhances spike-mediated ACE2 clustering in a cell-free system

**Figure 7:**
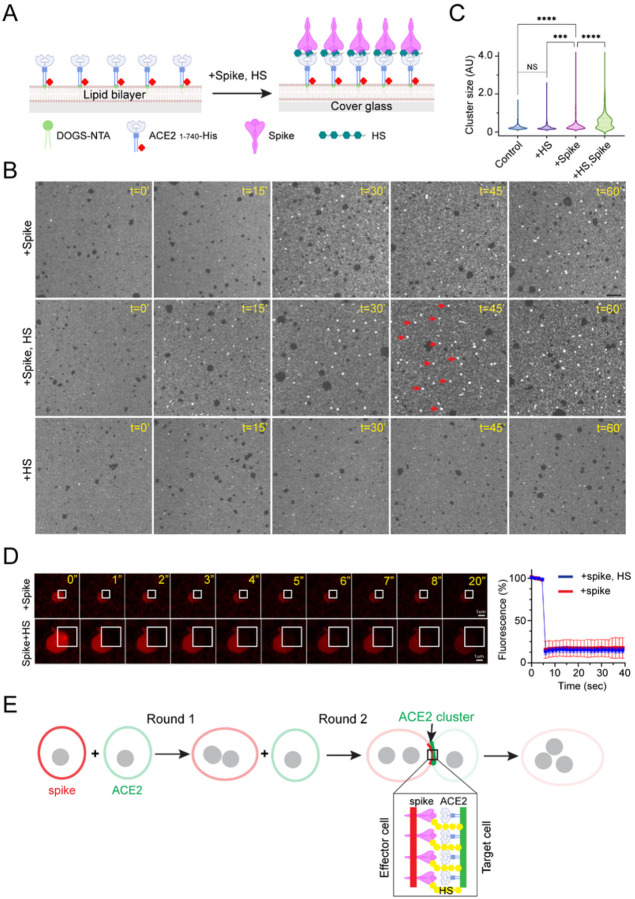
HS facilitates spike-induced ACE2 clustering in a cell free system (A) A schematic illustration of the *in vitro* ACE2-clustering assay. (B, C) HS stimulates ACE2 cluster formation in the presence of the spike. Fluorescence-labeled ACE2 1–740 anchored to lipid bilayers in an imaging chamber was incubated with spike (15 nM) and HS (2.5 μM) either individually or in combination at 37 °C for the indicated time points. Arrows indicate examples of ACE2 clusters. Scale bar, 10 μm. (C) Quantification of the ACE2 cluster size in (B). ****, p<0.0001 by one-way ANOVA. n=3 independent experiments. (D) ACE2 is immobile in *in vitro* formed clusters. The indicated area in an ACE2 cluster formed in the presence of spike (top panels) or spike combined with HS (bottom panels) were photobleached and imaged. The graph shows the GFP intensity in the indicated area over time. Scale bars, 1 μm. (E) HS-assisted ACE2 clustering helps overcome fusion-induced dilution of the spike, maintaining high levels of the fusogen at the fusion site.

We developed a cell-free receptor clustering assay to test whether HS directly promotes ACE2 clustering (Figure 7A). To this end, purified biotinylated ACE2 1–740 bearing a C-terminal 6xHis tag was labeled with Alexa_565_ and anchored to a cover glass coated with a lipid bilayer that consisted of 1-palmitoyl-2-oleoyl-sn-glycero-3-phosphocholine (POPC), 1,2-dioleoyl-sn—glycero-3-phosphoethanolamine-N-[methoxy(polyethylene glycol)-5000] (PEG-5000 PE), and 1, 2-dioleoyl-sn-glycero-3-[(N-(5-amino-1-carboxypentyl)iminodiacetic acid)succinyl] (DOGS-NTA). This protein contains just the ACE2 ECD and the conserved LS. In our method, the protein is anchored on the lipid bilayer in a highly mobile state, and importantly, with a topology mimicking membrane-anchored full-length ACE2. When membrane-anchored ACE2 1–740 was incubated with recombinant spike trimer, round-shaped bright speckles were formed due to ACE2 clustering, while incubating with buffer or HS alone did not induce ACE2 clustering. Consistent with our model, ACE2 clustering with spike alone was inefficient, but when HS and spike were added together, ACE2 cluster formation was significantly accelerated (Figure 7B, C). Like ACE2 clusters in cells, ACE2 in *in vitro*-assembled clusters was also immobile, as demonstrated by FRAP (Figure 7D). These results suggest that HS directly promotes ACE2 clustering in the presence of the spike.

Surprisingly, adding PIXN did not abolish spike/HS-induced ACE2 clustering. However, under this condition, ACE2 clustering generated mostly elongated laments (Figure S7), suggesting that PIXN alters the mode of ACE2 self-assembly. The differential effect of PIXN on ACE2 clustering in cells vs. *in vitro* suggests that additional factors modulate ACE2 self-interaction at the cell surface, preventing filament-like ACE2 clusters in the presence of PIXN.

## Discussion

The cause of clinical heterogeneity among SARS-CoV-2-infected patients is unclear, but it is suggested that spike-induced cell-cell fusion may be a contributor to inflammation, thrombosis, or lymphopenia observed in severe COVID-19 patients because multi-nuclei-containing syncytia were often seen in damaged lung tissues from posthumous COVID-19 organs^25–27^, and because spike-induced syncytia can rapidly internalize lymphocytes, resulting in a unique cell-in-cell structure seen in COVID-19 tissues but not in other types of pneumonia^25^. When expressed in neurons, spike can even drive the fusion of neurons with neurons or glia, which might contribute to the neurologic symptoms associated with long COVID-19^36^. Furthermore, spike-induced cell-cell fusion may contribute to viral transmission. Since viruses do not need to exit the infected cells, this transmission route allows the virus to escape immune surveillance, particularly antibody-mediated neutralization^28,29^. Intriguingly, spikes from SARS-CoV-2 variants of concern have different fusogenic activities with the Delta spike being the most potent fusogen. Coincidentally, studies have associated the emergence of the Delta variant with an increased risk of COVID-19-related hospitalization^37,38^.

*In vitro*, spike’s fusion-inducing activity can sustain over multiple rounds of fusion reactions despite the fact that the spike concentration at the cell surface is exponentially reduced after each round of fusion. How can the SARS-CoV-2 spike be so efficient at inducing membrane fusion? Given the fast fusion reaction, new protein synthesis is likely unable to compensate for the rapid reduction of the surface spike. Our data support a model in which HS facilitates an allosteric conformational change in spike-bound ACE2, allowing a conserved linker to promote ACE2 clustering. When clustered ACE2 engages spike on the opposite membrane, these multivalent protein interactions also concentrate the spike (Figure 5C) while bringing the effector and acceptor membranes together to form the synapse-like structure in preparation for membrane fusion. This model would explain the potent fusogenic activity of the spike despite the constant reduction of its concentration at the cell surface due to cell-cell fusion and other turnover mechanisms (Figure 7E).

Intriguingly, our results suggest that HS functions predominantly on the ACE2-containing acceptor membrane because HS on spike-containing membranes fails to compensate for the loss of HS on the ACE2 cells. Thus, HS might interact with the spike via a specific configuration not achievable when positioned *in cis* to the spike. Furthermore, free HS can promote ACE2 clustering and membrane fusion when added to the medium at high concentrations, suggesting that membrane association may increase local HS concentration and thus enhance its affinity to the spike. Consistent with this idea, HSPG antibody staining did detect HS in small puncta on the cell surface^9^.

Our study establishes PIXN and MTAN as HS-binding drugs targeting specific sulfate groups in HS. Although both drugs were initially reported as DNA topoisomerase inhibitors^39^ and had been evaluated extensively in clinics to treat various tumors^40,41^, PIXN has an overall improved clinical safety profile^31^. This fact, combined with the observed *in vivo* anti-SARS-CoV-2 activity, suggests further testing the antiviral activity of PIXN or other HS inhibitors in clinical trials for patients with severe COVID-19 symptoms.

Spike-induced membrane contacts are morphologically similar to previously reported immune synapses. Both immune and spike-induced synapses are enriched in actin laments, organized into ring-shaped structures surrounding the juxtaposed membranes. The dynamic movement of actin laments may provide a mechanical force that drives the dilation of the fusion pore during cell-cell fusion. Interestingly, in T cell receptor (TCR)-mediated immune synapses, ligand-receptor interaction is accompanied by the formation of large TCR clusters analogous to the ACE2 clusters observed in this study^42^. Thus, an intriguing question is whether HS may play a more general role in shaping the protein interaction networks at the cell surface during cell-cell communications and cell signaling. Indeed, HS was reported to regulate the activation of many signaling receptors, including the FGF receptor, Wnt, and Hedgehog receptor^43^. Whether these receptors form super-clusters in an HS-regulated manner upon ligand binding awaits future investigations.

## Online Methods

### Chemical Synthesis

#### General Procedures.

All commercial reagents were used as provided unless otherwise indicated. An anhydrous solvent dispensing system (J. C. Meyer) using 2 packed columns of neutral alumina was used for drying THF, Et_2_O, and CH_2_Cl_2,_ whereas 2 packed columns of molecular sieves were used to dry DMF. Solvents were dispensed under argon. Flash chromatography was performed with RediSep R_f_ silica gel columns on a Teledyne ISCO CombiFlash^®^ R_f_ system using the solvents as indicated. Nuclear magnetic resonance spectra were recorded on a Varian 600 MHz or Bruker 400 MHz spectrometer with Me_4_Si or signals from residual solvent as the internal standard for ^1^H or ^13^C. Chemical shifts are reported in ppm, and signals are described as s (singlet), d (doublet), t (triplet), q (quartet), m (multiplet), and br s (broad singlet). Values given for coupling constants are first order. High resolution mass spectra were recorded on an Agilent TOF II TOF/MS instrument equipped with either an ESI or APCI interface at the Center for Drug Design, University of Minnesota (Minneapolis, MN, USA).

#### LC-1541 (PIXN).

To compound 6,9-difluorobenzo[g]isoquinoline-5,10-dione (220 mg, 0.89 mmol, 1.0 eq.) in THF (20 mL) under argon atmosphere was added ethane-1,2-diamine (0.6 mL, 8.97 mmol, 10.0 eq.). The resultant solution was stirred at 50 °C for 24 h. The mixture was diluted with methanol (10 mL) and the solvents were removed under vacuum. The crude product was purified by column chromatography (30% MeOH: NH_4_OH (9:1) in CH_2_Cl_2_) to give compound LC-1541 as a blue gum (95 mg, 56%). ^1^H NMR (600 MHz, CD_3_OD) δ 9.44 (s, 1H), 8.88 (d, *J* = 5.2 Hz, 1H), 8.14 (d, *J* = 5.2 Hz, 1H), 7.52 (d, *J* = 4.2 Hz, 2H), 3.71 (td, *J* = 6.4, 2.0 Hz, 4H), 3.13 (q, *J* = 6.0 Hz, 4H). HRMS (ESI^+^): m/z calcd for C_17_H_20_N_5_O_2_ [M+H]^+^ 326.1612, found 326.1602.

The following compounds were prepared in a fashion similar to the one described for LC-1541.

#### LC1539.

^1^H NMR (600 MHz, DMSO-*d*_6_) δ 13.49 (s, 2H), 10.45 (t, *J* = 6.5 Hz, 2H), 7.67 (s, 2H), 7.22 (s, 2H), 3.82 (q, *J* = 6.5 Hz, 4H), 3.06 (d, *J* = 8.8 Hz, 4H). HRMS (ESI^+^): m/z calcd for C_18_H_21_N_4_O_4_ [M+H]^+^ 357.1557, found 357.1559.

#### LC1540.

^1^H NMR (600 MHz, CD_3_OD) δ 9.04 (d, *J* = 1.8 Hz, 1H), 8.67 (d, *J* = 5.0 Hz, 1H), 7.74 (d, *J* = 4.9 Hz, 1H), 6.99 (dd, *J* = 4.2, 2.3 Hz, 2H), 3.75 (q, *J* = 3.5 Hz, 4H), 3.45 (dt, *J* = 13.0, 6.5 Hz, 4H), 2.95 (td, *J* = 6.5, 3.6 Hz, 4H), 2.87 (td, *J* = 5.5, 1.6 Hz, 4H). HRMS (ESI^+^): m/z calcd for C21H_28_N_5_O_4_ [M+H]_+_ 414.2136, found 414.2132.

#### LC1542.

^1^H NMR (600 MHz, CD_3_OD) δ 7.38 (s, 2H), 7.32 (s, 2H), 3.92 (s, 6H), 3.70 (t, *J* = 5.5 Hz, 4H), 3.55 (t, *J* = 6.4 Hz, 4H), 2.98 (t, *J* = 6.5 Hz, 4H), 2.83 (t, *J* = 5.5 Hz, 4H). HRMS (ESI^+^): m/z calcd for C_24_H_33_N_4_O_6_ [M+H]^+^ 473.2395, found 473.2395.

#### LC1553.

^1^H NMR (600 MHz, CD_3_OD) δ 7.27 (d, *J* = 9.2 Hz, 1H), 7.17 (d, *J* = 9.7 Hz, 1H), 7.07 (d, *J* = 9.8 Hz, 1H), 7.02 (d, *J* = 9.1 Hz, 1H), 3.87 (s, 3H), 3.71 (dt, *J* = 7.7, 5.5 Hz, 4H), 3.49 (t, *J* = 6.5 Hz, 2H), 3.44 (t, *J* = 6.5 Hz, 2H), 2.97 (t, *J* = 6.5 Hz, 2H), 2.93 (t, *J* = 6.5 Hz, 2H), 2.83 (dt, *J* = 11.6, 5.6 Hz, 4H). HRMS (ESI^+^): m/z calcd for C_23_H_31_N_4_O_6_ [M+H]^+^ 459.2238, found 459.2233.

#### LC1519.

^1^H NMR (600 MHz, CD_3_OD) δ 6.81 (s, 2H), 6.75 (s, 2H), 3.70 (t, *J* = 4.5 Hz, 4H), 3.65 (t, *J* = 5.1 Hz, 4H), 3.58 (t, *J* = 4.5 Hz, 4H), 3.34−3.26 (m, 4H), 2.91−2.83 (m, 8H). HRMS (ESI^+^): m/z calcd for C_26_H_37_N_4_O_8_ [M+H]^+^ 533.2606, found 533.2601.

#### LC1520.

^1^H NMR (600 MHz, CD_3_OD) δ 6.91 (s, 2H), 6.90 (s, 2H), 3.69−3.61 (m, 16H), 3.54 (t, *J* = 4.8 Hz, 4H), 3.37 (t, *J* = 6.6 Hz, 4H), 2.92−2.84 (m, 8H). HRMS (ESI^+^): m/z calcd for C_30_H_45_N_4_O_10_ [M+H]^+^ 621.3130, found 621.3136.

#### LC1521.

^1^H NMR (600 MHz, CD_3_OD) δ 7.02 (s, 2H), 6.95 (s, 2H), 3.68−3.62 (m, 16H), 3.62−3.57 (m, 8H), 3.52 (t, *J* = 4.5 Hz, 4H), 3.44 (t, *J* = 6.9 Hz, 4H), 2.92 (t, *J* = 6.6 Hz, 4H), 2.88 (t, *J* = 5.1 Hz, 4H). HRMS (ESI^+^): m/z calcd for C_34_H_53_N_4_O_12_ [M+H]^+^ 709.3654, found 709.3648.

### Chemoenzymatic synthesis of HS 6-mers

A total of seven 6-mers were synthesized in this study using the chemoenzymatic synthetic approach^44^. These 6-mers are differed in the number of sulfo groups as well the presence or absences of 2-*O*-sulfated iduronic acid (IdoA2S) residue. All synthesis was initiated from glucuronide para-nitrophenyl (GlcA-pNP), which is commercially available (Carbosyn). To synthesize the 6-mers without an IdoA2S residue, the synthesis involved the use of heparosan synthase 2 from Pasteurella multocida (pmHS2) and UDP-GlcNAc (or UDP-GlcNTFA, NTFA represents *N*-trifiuoroacetylated glucosamine was incubated with pmHS2 (30 mg) and UDP-GlcNAc (3 mM) in a 100 mL of the reaction buffer containing 25 mM Tris-HCl (pH 7.2), 5 mM MnCl_2_. The reaction was incubated at 37 °C overnight, and the 2-mer product was purified by a C-18 reverse phase column. The 2-mer product was further elongated to 3-mer in the 100 mL reaction buffer (pH 7.2) containing UDP-GlcA and purified on a C-18 column. The elongation and purification steps were repeated until the desired 6-mer was achieved. The final compound was confirmed for structural identity with Mass Spec and purity was checked with analytical HPLC. The pmHS2 enzyme, UDP-GlcA and UDP-GlcNTFA were made according to the protocol described in a prior publication^45,46^. Additional modification steps, including N-sulfation, 6-O-sulfation were completed using N-sulfotransferase and 6-*O*-sulfotransferase isoform 3, respectively^46^. To install an IdoA2S residue, 2-O-sulfotransferase and C5-pimerase were employed. The 3-O-sulfation to prepare NS2S6S3S 6-mer, 3-O-sulfotransferanse was used. The purity of the products was confirmed by high resolution anion exchange HPLC, and the molecular weight (MW) was determined by electrospray ionization mass spectrometry. As shown below, the purity of the 6-mers was in the range of 92% to 99%, and the measured MW was very close to the calculated MW (Calc MW).

**Table:** 

ID	Abbreviated sequence	Calc MW	Measured MW	Purity by HPLC
OS	GlcNAc-GlcA-GlcNAc-GlcA-GlcNAc-GlcA-pNP*	1277	1277	99%
NS	GlcNS-GlcA-GlcNS-GlcA-GlcNS-GlcA-pNP	1391	1391	99%
2S	GlcNS-GlcA-GlcNS-ldoA2S-GlcNS-GlcA-pNP	1471	1470	97%
NS6S	GlcNS6S-GlcA-GlcNS6S-GlcA-GlcNS6S-GlcA-pNP	1631	1630	94%
NS2S6S3S	GlcNS6S-GlcA-GlcNS6S3S-ldoA2S-GlcNS6S-GlcA-pNP	1791	1791	92%
NS6S(6)	GlcNAc6S-GlcA-GlcNAc6S-GlcA-GlcNS6S-GlcA-pNP	1555	1554	91%
NS6S(7)	GlcNS6S-GlcA-GlcNAc6S-GlcA-GlcNAc6S-GlcA-pNP	1555	1554	99%

* pNP refers to *para*-nitrophenyl group

### Cell culture and virus infection

293T cells stably expressing GFP-tagged human ACE2 (ACE2-GFP) were reported previously^9^. To make SLC35B2 KO 293T cells expressing ACE2-GFP, SLC35B2 CRISPR KO cells^30^ were transfecting cells with pCMV-ACE2-GFP (Codex). GFP-positive cells were sorted by FACS after neomycin (1 mg/mL) selection for 1 week. These cells were maintained in Dulbecco’s modified Eagle’s medium (DMEM) supplemented with 10% fetal bovine serum (FBS), 1% penicillin/streptomycin.

Vero TMPRSS2-E6 (TE6), a Vero E6-based cell line stably transfected with human TMPRSS2 was obtained from BPS Bioscience (San Diego, CA) and was maintained in Gibco high-glucose Dulbecco’s modified Eagle’s medium (DMEM) supplemented with 10% fetal bovine serum (FBS), 1% penicillin/streptomycin, 10 mM HEPES pH 7.3, 1% Na pyruvate plus 3 μg/mL of Puromycin in a 37 °C incubator supplemented with 5% CO2. Vero E6-TMPRSS2-T2A-ACE2 (TA6), a Vero E6 cell line overexpressing both human TMPRSS2 and human ACE2 was obtained from BEI Resources (Manassas, VA) and was maintained in Gibco high-glucose Dulbecco’s modified Eagle’s medium (DMEM) supplemented with 10% fetal bovine serum (FBS), 1% penicillin/streptomycin, 10 mM HEPES pH 7.3 plus 10 μg/mL of Puromycin in a 37 °C incubator supplemented with 5% CO2.

The seeds of SARS-CoV-2 clinical isolates — USA-WA1/2020 and the Delta variant were obtained through BEI Resources (Manassas, VA). All seed viruses were amplified in TE6 cells in the infection medium (DMEM supplemented with 3% FBS) at 37 °C, 5% CO2 for 3 days. Amplified viruses were aliquoted and stored in a secured −80 °C freezer until use. Virus titers were titrated in TE6 cells using an ELISA-based 50% tissue culture infectious dose (TCID_50_) method^47,48^.

### Infection with live SARS-CoV-2 in cultured cells

Vero TA6 cells were pre-seeded into 8-chamber slides (ibidi #80800) overnight. The next day after the aspiration of the growth media, cells were pre-treated with PIXN, MTAN, or DMSO diluted in the infection medium at 37°C for 30 mins. Then, without removing the compounds, cells were infected with USA-WA1/2020 or Delta variant (MOI of 0.1 or 0.5) at 37 °C, 5% CO2 for another four 4 h before fixation and staining. For heparinase I/III treatment, cells were treated with a Heparinase I and III mixture that contains 180 μL DMEM medium, 20 μL 10x digestion buffer (NEB), 1.6 μL heparinase I, 4.0 μL heparinase II at 37 °C for 3 hours. Treated cells were washed once with the medium and then subject to infection or spike-induced cell-cell fusion. Cells were fixed with 4% paraformaldehyde in Phosphate Buffer Saline (PBS) for 10 min followed by 4 washes with PBS before antibody staining. To confirm the removal of HS, we incubated cells with recombinant CFP+ at ~100 nM on ice for 10 min before fixation and imaging.

### Infection with live SARS-CoV-2 in mice

B6.Cg-Tg(K18-ACE2)2Prlmn/J (K18-hACE2) transgenic mice (JAX Stock No. 034860)^33^ were bred at FDA White Oak Vivarium and were individually genotyping-confirmed (Transnetyx) before experiments. Age-matched male and female K18-hACE2 adult mice (the male and female ratio was approximately 1:1) at 12–16 weeks of age were injected intraperitoneally with 0.1 mL/mouse of PIXN at a final dose of 50 or 100 mg/kg, respectively. PIXN was dissolved in sterile PBS (pH 7.4) containing 1% of Penicillin/Streptomycin. K18-hACE2 mice receiving 0.1 mL/mouse of sterile PBS (pH7.4) containing 1% of Penicillin/Streptomycin served as controls. Two hours after injection, mice were inoculated intranasally with 1000 TCID_50_/50 μL/mouse of live infectious USA-WA1/2020. Three days after infection, mice were euthanized, and whole lungs were harvested for viral load determination. Lungs were homogenized, and total RNA was extracted using the RNeasy Plus Mini Kit. The copies of the viral nucleocapsid (N) gene in homogenized lung tissues were amplified using the High-Capacity cDNA Reverse Transcription Kit and QuantiNova SYBR Green PCR kit in combination with 500 nM of 2019-nCoV RUO Kit according to the following program: 95 °C for 120 s, 95 °C for 5 s and 60 °C for 18 s (50 cycles) 1,3. A value of 1 was assigned if gene copies were below the detection limits. The mouse infection experiments with live SARS-CoV-2 were performed in an FDA Animal Biosafety Level-3 (ABSL-3) laboratory equipped with advanced access control devices and by trained personnel equipped with powered air-purifying respirators.

### Pseudoviral particle entry assay

HEK293T-ACE2-GFP cells were seeded in white, transparent bottom 96-well microplates at 20,000 cells per well in 100 μL growth medium and incubated at 37 °C with 5% CO2 overnight (~16 h). The growth medium was carefully removed, and 50 μL PP or PP-containing compounds were added to each well. The plates were then spinoculated by centrifugation at 1500 rpm (453× g) for 45 min and incubated for 24 h (48 h for Calu-3 cells) at 37 °C, 5% CO2 to allow cell entry of PP and the expression of luciferase. After incubation, the supernatant was carefully removed. Then 50 μL/well of Bright-Glo luciferase detection reagent (Promega) was added to assay plates and incubated for 5 min at room temperature. The luminescence signal was measured by a Victor 1420 plate reader (PerkinElmer). For ACE2-GFP cells, the GFP signal was also determined by the plate reader. Data were normalized with wells containing PP but no compound as 100%, wells mock-treated with phosphate buffer saline (PBS) as 0%, and the ratio of luciferase to the corresponding GFP intensity was calculated.

### SARS-CoV-2 infection in a 3D EpiAirway model

Human bronchial epithelial cells (HBEC’s 3D-EpiAirway^™^) were seeded into culture inserts for 6-well plates one day before viral infection. Before adding drugs or virus, accumulated mucus from the tissue surface was removed by gently rinsing the apical surface twice with 400 μL TEER buffer. All fluids from the tissue surface were carefully removed to leave the apical surface exposed to the air. MTAN was diluted into the assay medium and placed at room temperature before co-treatment with a virus (MOI of 0.1) onto the apical and basal layers for one h. Following one h treatment, the virus was removed from the apical layer. The basolateral medium was replaced with fresh maintenance medium and compound at 24 h, 48 h, and 72 h post-infection.

At 24 hours and 96 hours post-infection, the apical layer was washed with 0.4 mL of the TEER buffer (PBS with Mg2+ and Ca2+). The washes were combined and aliquoted into separate microfuge tubes (1.5 mL). Eight-fold serial dilutions of apical layer supernatant sample concentrations were added to 96-well assay plates containing Vero E6 cells (20,000/well). The plates were incubated at 37 °C, 5% CO2, and 95% relative humidity. Following three days (72 ± 4 h) incubation, the plates were stained with crystal violet to measure the cytopathic effect (CPE). Virus titers were calculated using the method of Reed and Muench (Reed et al., 1938). The TCID_50_ values were determined from duplicate samples.

### LDH assay

Medium from the basolateral layer of the 3-D tissue culture inserts was removed 24- and 96-h post-infection and diluted in an LDH Storage Buffer per the manufacturer’s instructions (LDH-Glo Cytotoxicity Assay, Promega). Samples (5 μL) were further diluted with the LDH Buffer (95 μL) and incubated with an equal volume of LDH Detection Reagent. Luminescence was recorded after 60 min incubation at room temperature. As a negative control, we included a no-cell sample in determining the culture medium background. We used tissues treated with the apoptosis-inducing drug bleomycin as a positive control.

### ATP-based cytotoxicity assay

HEK293T-ACE2-GFP cells were seeded in a white, transparent bottom 96-well microplate (Thermo Fisher Scientific) at 20,000 cells per well in 100 μL growth medium and incubated at 37 °C with 5% CO2 overnight (~16 h). The growth medium was carefully removed, and 100 μL medium with compounds was added into each well. The plates were then incubated at 37 °C for 24 h (48 h for Calu-3 cells) at 37 °C 5% CO2. After incubation, 50 μL/well of ATPLite (PerkinElmer) was added to assay plates and incubated for 15 min at room temperature. The luminescence signal was measured using a Victor plate reader (PerkinElmer). Data were normalized with wells containing cells but no compound as 100% and wells containing media-only as 0 %.

### NMR study

NMR spectra were recorded at 313 K with a Bruker Avance III spectrometer operating at 850 MHz and equipped with a high sensitivity 5 mm TCI cryoprobe. Samples were lyophilized twice to remove residual solvents and were then dissolved in D2O (99.996%, Sigma, Co.) and placed in 5 mm NMR tubes. For the experiments involving free ligands, the samples were prepared to obtain a final concentration of 1.2×10–3 M by dissolving hexasaccharide (1 mg) and Pixantrone (0.2 mg) in D2O. Proton spectra were recorded using water presaturation with a recycle delay of 10 seconds and 24 scans.

HSQC (heteronuclear single quantum coherence) experiments were performed in phase-sensitive mode with Echo/Antiecho-TPPI gradient selection using decoupling during acquisition and multiplicity editing during the selection step.

Thirty-two dummy scans and 20 scans with decoupling during the acquisition period with 1.5 seconds relaxation delay were accumulated, using a matrix size of 2048×256 datapoints.

Bidimensional HSQC-TOCSY data were acquired by using 20 scans per increment using a 2048×256 datapoints matrix with zero-filling in F1 to 2048×2048 points. 1H-13C HSQC-TOCSY were performed using 1.5 seconds relaxation delay and 100 msec mixing time.

For NOE experiments, the samples were prepared by dissolving hexasaccharide (1 mg) and Pixantrone (0.1 mg) in D2O, reaching a molar ratio of hexasaccharide/Pixantrone 2:1. NOESY experiments were performed at 313 K. A total of 24 scans was collected for each free induction decay (matrix 2048×256 points), the data were zero-filled to 2048×2048 points before Fourier transformation, and mixing time values of 120 ms was used.

### Drug and protein binding studies

HS or HS 6-mers of different concentrations were added to MTAN or PIXN (50uM). After a brief incubation, absorbance was measured from 500 nm to 700 nm by Nanodrop 2000 (Thermo Fisher Scientific). The change in OD650 was determined and fitted into a binding curve using GraphPrism 9.0.

To study the interaction of spike with ACE2 *in vitro*, we used spike (200 ng/well) in phosphate buffer saline (PBS) to treat a high binding 96 well plate at 4 °C for 15 h. The spike-coated plate was washed with PBS once and then incubated with the TMS buffer containing 4% bovine serum albumin (BSA), 20 mM Tris-HCl, 7.4, 150 mM NaCl, 2mM MgCl2 2 mM CaCl2 for three h at 37 °C. The plate was washed once with a BSA-free TMS buffer and then incubated for one h with heparin (5 μM) together with PIXN (20 μM) in the TMLS buffer containing 20 mM Tris-HCl, 7.4, 50 mM NaCl, 2mM MgCl2 2 mM CaCl2, 1% BSA to allow heparin binding to the spike. The plate was washed with the TMLS buffer once and then incubated with ACE2 recombinant protein bearing an hFC tag at various concentrations at room temperature for 1 h in the TMLS buffer. The plate was washed three times with the TMS buffer and then incubated with HRP-conjugated protein A in the TMS buffer for 1 h. The plate was washed four times and then developed with TMB turbo substrate for 5–10min before the addition of 1 M sulfuric acid to quench the reactions. The absorbance was measured at 450 nm.

### Spike- and ACE2-mediated cell fusion assay

293T cells expressing spike protein and mCherry were generated by transfecting cells in a 3.5 mm culture dish with 2.0 μg of pcDNA3.1-SARS-CoV-2-Spike (From the Delta variant) and pLV-mCherry at 10:1 ratio for 24 h. Acceptor cells are 293T cells stably expressing ACE2-GFP reported previously (Zhang *et al*., 2020a). ACE2-GFP cells were seeded in bronectin-coated 8-well Ibidi glass-bottom chambers at 50,000/well on day 1. Cell-cell fusion was conducted 48 hours later. For each fusion reaction, 50,000 spike/mCherry-transfected cells (effector cells) were added to the imaging chamber. Live cell 4D imaging was initiated ~1 minute after adding the effector cells. The fusion reactions were stopped for immunostaining experiments by incubating cells with a fixing buffer containing 4% paraformaldehyde in PBS for 15 min at room temperature. Cells were then stained with a Hoechst 33,342 staining solution to label the nuclei or with primary and secondary antibodies diluted in a PBS-based staining buffer containing 10% FBS and 0.2% Saponin.

### Transmission electron microscopy

Spike/mCherry donor cells and ACE2-GFP acceptor cells were mixed at 1:1 ratio and incubated in suspension at 37 °C for 12 min to form spike- and ACE2-induced membrane synapses. Cells were then fixed in a mixture of 2.5% glutaraldehyde and 1% paraformaldehyde in 0.1M phosphate buffer, pH 7.4, for 90 minutes at room temperature. Subsequently, samples were washed three times for ten minutes each in 0.1M sodium cacodylate buffer, pH 7.4, before being post-fixed in 1% OsO4 and 1.5% K3Fe(CN)6 in 0.1M cacodylate buffer for 60 minutes on ice. Next, samples were rinsed and washed two times for ten minutes each in water and incubated with 1% uranyl acetate overnight at 4 °C. The following day samples were rinsed and washed in water for ten minutes and gradually dehydrated through a graded ethanol series followed by propylene oxide. Samples were then infiltrated in a gradient mix of propylene oxide and resin (Embed 812 resin) before being infiltrated with three changes of pure resin and embedded in 100% resin and baked at 60°C for 48 hours. Ultrathin sections (65 nm) were cut on an ultramicrotome (Leica EM UT7), and digital micrographs were acquired on JOEL JEM 1200 EXII operating at 80 Kv and equipped with an AMT XR-60 digital camera.

### *In vitro* ACE2 clustering assay

To prepare small unilateral vesicles (SUV), we used glass syringes to prepare a DOGS-NTA lipid mixture in a glass vial as follows: rinse a glass vial with chloroform, and then add ~1 mL of chloroform plus individual lipids (POPC, 2.98mg; DOGS-NTA, 0.085mg, PEG-5000 PE, 0.023 mg). The lipid mixture was dried with a stable flow of nitrogen and then in a vacuum desiccator for 2 h. Resuspend the dried lipids in 1.5 mL of PBS and vortex. Transfer the resuspension into two 1.5 mL conical microcentrifuge tubes. Freeze the lipid resuspension in liquid nitrogen and thaw immediately in a water bath at room temperature. Repeat the freeze-thaw for 30 cycles. The cloudy solution will become clear over the freeze-thaw cycles. The lipid resuspensions were centrifuged at 22,000 × *g* for 45 min at 4 °C. The SUV-containing supernatant was collected in a clean tube.

Ibidi u-Slide 8 well Glass Bottom chambers were soaked in 5% Hellmanex III for 24 h (pre-heated to 50 °C) overnight, rinsed extensively with ultrapure water, and air dried. The glass surface was then treated with NaOH 1M for 1 h at 50 °C. Treated chambers were rinsed with ultrapure water and washed 3 times with the Basic buffer (HEPES pH 7.3 50mM, NaCl 150mM). Each well was treated with 200 μL Basic buffer containing 7uL SUV at 37 °C for 1 h followed by three washes with the Basic buffer. After the last wash, we blocked the lipid-coated surface with 200 μL Clustering Buffer (Basic buffer plus BSA 1 mg/mL) at 37 °C for 20 min. Add His8-tagged, Alexa555-labeled ACE2 1–740 (~50 nM) in the Clustering Buffer to each well and incubate the chamber at 29 °C for 1 h to allow His-tagged ACE2 to attach to the lipid bilayer. Wash each well three times with the Signaling Buffer (HEPES pH 7.3 50mM, NaCl 50mM). At this point, determine the mobility of ACE2 by Fluorescence Recovery After Photobleaching (FRAP). Add the spike in the Clustering Buffer at a final concentration of 14 nM in the presence or absence of HS. ACE2 clustering was imaged at the indicated time point by a Nikon CSU-W1 SoRa microscope equipped with a temperature control enclosure.

### Imaging processing and statistical data analysis

Fluorescence confocal images were acquired by a Nikon CSU-W1 SoRa microscope equipped with a temperature control enclosure and a CO_2_ control. 3D or 4D image reconstructions and analyses were done by Imaris software (Licensed to NIH). Fluorescence intensity was analyzed by open-source Fiji software. To this end, images were converted to individual channels, and regions of interest were drawn for measurement. Statistical analyses were performed using either Excel or GraphPad Prism 9.0. *P* values were calculated by Student’s *t*-test using Excel or one-way ANOVA by GraphPad Prism 9.0. Linear curve fitting, nonlinear curve fitting, and IC50 calculation were done with GraphPad Prism 9.0. For nonlinear fitting, the inhibitor vs. response -variable slope model or the exponential decay model was used. Images were prepared by Photoshop and Illustrator (Adobe). Data processing and reporting are adherent to the community standards.
